# The Bigger the Better? Analysis of Surgical Complications and Outcome of the Retrosigmoid Approach in 449 Oncological Cases

**DOI:** 10.3389/fonc.2022.938703

**Published:** 2022-07-05

**Authors:** Amir Kaywan Aftahy, Ann-Kathrin Jörger, Sandra Hillebrand, Felix N. Harder, Benedikt Wiestler, Denise Bernhardt, Stephanie E. Combs, Bernhard Meyer, Chiara Negwer, Jens Gempt

**Affiliations:** ^1^ School of Medicine, Technical University Munich, Department of Neurosurgery, Klinikum rechts der Isar, Munich, Germany; ^2^ School of Medicine, Technical University Munich, Department of Radiology, Klinikum rechts der Isar, Munich, Germany; ^3^ School of Medicine, Technical University Munich, Department of Neuroradiology, Klinikum rechts der Isar, Munich, Germany; ^4^ School of Medicine, Technical University Munich, Department of Radiation Oncology, Klinikum rechts der Isar, Munich, Germany; ^5^ German Cancer Consortium (DKTK), Partner Site Munich, Munich, Germany; ^6^ Institute of Innovative Radiotherapy (iRT), Department of Radiation Sciences (DRS), Helmholtz Zentrum Munich, Munich, Germany

**Keywords:** neurooncology, operative technique, retrosigmoid approach, surgical technical improvement, skull base surgery

## Abstract

**Introduction:**

Exposure of the posterior skull base and the cerebellopontine angle is challenging due to important neurovascular structures. The retrosigmoid approach (RSA) has become the standard method used in surgery. We report our experiences with RSAs regarding technical obstacles, complications, and approach-related outcomes.

**Materials and Methods:**

We performed a retrospective chart review at a tertiary neurosurgical center between January 2007 and September 2020. We included all patients undergoing surgery for oncologic lesions through RSAs, concentrating on surgical technique, postoperative outcome, and complications.

**Results:**

A total of 449 RSAs were included. The median age at the time of surgery was 58 years; 168 (37.4%) were male and 281 (62.6%) were female. The median approach surface was 7.8 cm^2^. The median tumor volume was 5.9 cm^3^. The median Clavien–Dindo grade was 2, the total complication rate was 28.7%, and gross total resection (GTR) was 78.8%. Findings revealed that tumor volume had no significant impact on postoperative complications in general (p = 0.086) but had a significant impact on postoperative hemorrhage (p = 0.037) and hydrocephalus (p = 0.019). Tumor volume was significant for several preoperative symptoms (p < 0.001). The extent of the approach had no significant impact on complications in general (p = 0.120) but was significant regarding postoperative cerebrospinal fluid (CSF) leaks (p = 0.008). Craniotomy size was not significant regarding GTR (p = 0.178); GTR rate just missed significant correlation with tumor volume (p = 0.056). However, in the case of vestibular schwannomas, the size of craniotomy was important for GTR (p = 0.041).

**Conclusion:**

Tumor volume has an important impact on preoperative symptoms as well as on postoperative complications. Although the extent of the craniotomy barely missed significance regarding GTR, a correlation can be assumed. Thus, the extent of craniotomy should be taken into presurgical consideration, especially in the case of postoperative CSF leaks. Regarding vestibular schwannomas, craniotomy size plays an important role in achieving satisfactory oncological outcomes. Different approaches should be selected where necessary regarding superior resection rates.

## Introduction

The cerebellopontine angle (CPA) harbors several pathologies, such as chordomas, chondrosarcomas, cholesterol granulomas, paraganglioma, schwannomas, and neurovascular compression syndromes (1–7).

The inframeatal region extends from the lower boundary of the internal auditory canal to the glossopharyngeal nerve’s exit zone and is bounded anteriorly by the petrous part of the internal carotid artery and medially by the petrous apex (2, 5, 8–10).

Surgical approaches to these areas, such as the petrosal approach or the transcochlear approach, jeopardize hearing and endanger the facial nerve *inter alia*. Furthermore, extensive bone drilling is associated with a high risk of a cerebrospinal fluid (CSF) leak (1, 11).

The retrosigmoid approach (RSA) is the standard approach regarding the posterior fossa and the CPA. Technical details and variations have already been discussed in detail (3, 9, 12–15).

This manuscript aims to share our experience with a large series of performed RSAs for different pathologies at a tertiary neurosurgical university center. By focusing on technical issues, this study aims to question the necessity of the extent of the approach and to highlight approach-related complications, surgical success, and the postoperative outcome to improve the effectivity and reduce perioperative morbidity.

## Materials and Methods

### Study Population and Clinical Parameters

We performed a non-interventional retrospective single-center study. Between January 2007 and September 2020, we screened the clinical documentation files and neuropathological records of patients who underwent surgery through an RSA.

We analyzed clinical patient files for neurological symptoms, Karnofsky Performance Status Scale (KPSS), postoperative new neurological deficits, postoperative complications, re-interventions, and adverse events according to the Clavien–Dindo Classification (CDC). Radiological outcome parameters consisted of anatomic location as well as the extent of resection defined by comparing pre- and postoperative 3.0-T cranial MRI. This was achieved using T1 ± contrast agent sequences by manual volumetric segmentation, using the Origin^®^ software (Brainlab, version 3.1, BrainlabAG, Munich, Germany). We used the patients’ existing MRIs for craniotomy measurement. To obtain a three-dimensional impression of the craniotomy defect, the defect was first marked as precisely as possible in two views—axial and coronal—using crosshairs consisting of two planes. In the sagittal view, the defect appears as an approximately three-dimensional impression, which a region of interest (ROI) area measurement tool could determine in the largest diameter.

Statistical analysis, including descriptive data analysis, was performed using IBM SPSS Statistics Version 26.0 (SPSS Inc., IBM Corp., Armonk, NY, USA). Data are shown as median and interquartile range or as mean and SD. Spearman’s non-parametric rank correlation test was used to examine relationships between the continuous variables. For categorical variables, unpaired Mann–Whitney U tests were used to compare two samples. Proportions and group differences were analyzed with the chi-square statistic or Fisher’s test if the sample size was insufficient. p ≤ 0.05 was considered significant.

### Retrosigmoid Approach

Analogous to the pterional approach for the anterior skull base, the RSA is the standard approach regarding the posterior fossa and the CPA. Technical details have already been discussed in detail (3, 9, 12, 13).

**Figure 1:**
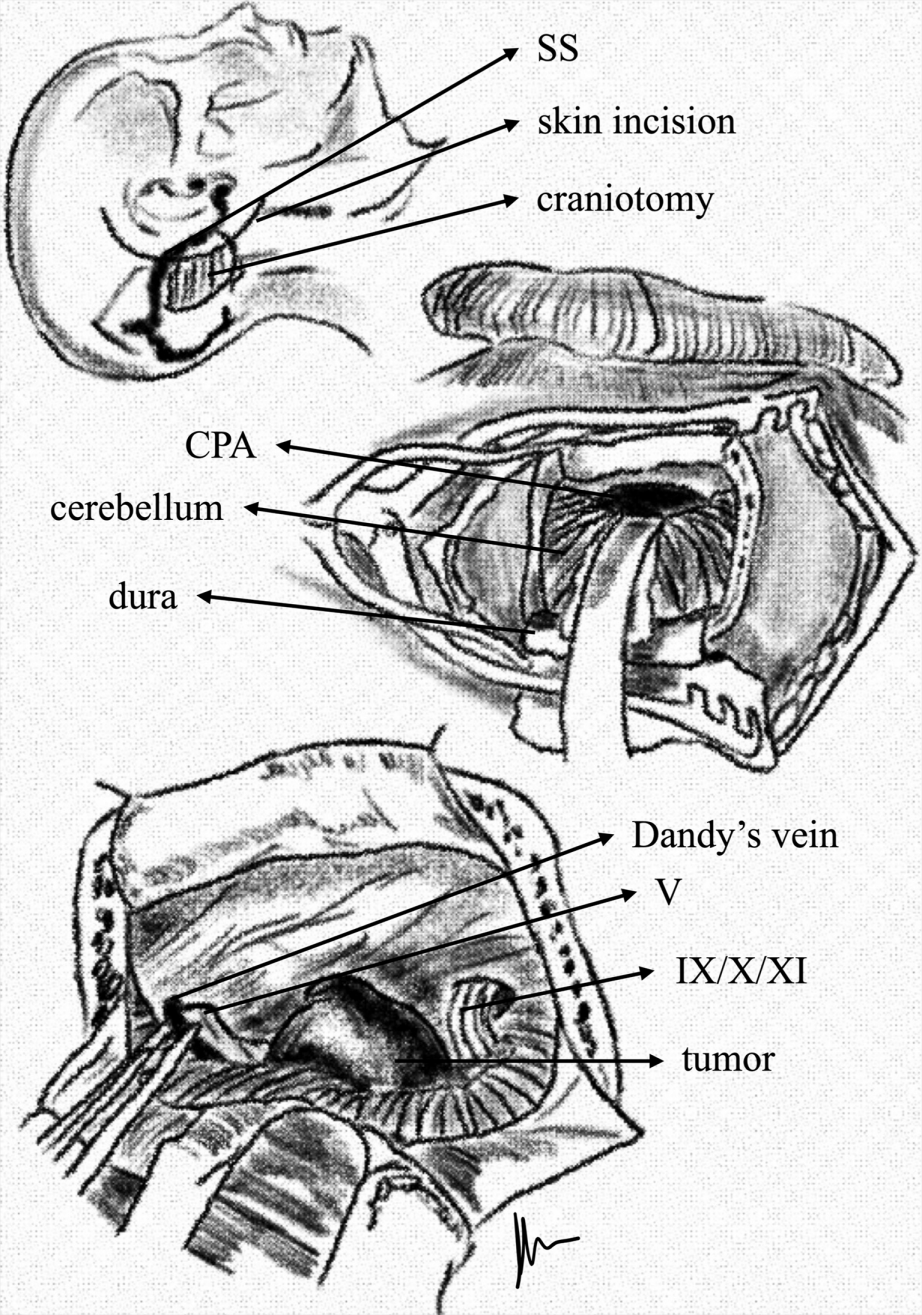
C-shaped incision used in the RSA. After craniotomy and retraction of the cerebellum, the tumor becomes visible within the cerebellopontine angle. The anterior edge of craniotomy is placed immediately behind the sigmoid sinus and just inferior to the lower margin of the transverse sinus.

We prefer a C-shaped skin incision. Several of the key steps in the RSA include the following (**Figure 1**): an osteoplastic or osteoclastic approach is performed, with the superior and anterior margins bordering the transverse and sigmoid sinuses, respectively; allow initial CSF release from the cerebellomedullary cistern to expose the CPA; separate the two layers of the arachnoid; provide significant decompression of the lesion/pathology before the dissection; and drill the posterior wall of the internal acoustic canal, if needed.

## Results

### Study Population

We included 449 RSAs between January 2007 and September 2020. The median age at the time of surgery was 58 years with 168 (37.4%) male and 281 (62.6%) female patients. Vertigo (50.2%), gait disturbance (24.6%), headache (21.4%), hypoacusia (43.1%), and facial nerve palsy (10.9%) were common preoperative symptoms. **Table 1** provides detailed demographic and clinical preoperative information, also separated for single pathology reflecting commonly known clinical symptoms of each (**Table 1**).

**Table 1:** Demographics, clinical presentation, and tumor histopathology.

		epidermoid cyst (N=15)		hemangioblastoma (N=9)		meningioma (N=157)		metastasis (N=49)		non-vestibular schwannomas (N=14)		others (N=25)		vestibular schwannoma (N=180)		total (N=449)	
**age (years)**	mean	50.2		49.33		60.27		62.67		56.43			51.44		54.5		57.07
	SD	11.827		19.281		14.081		13.375		18.308			18.597		15.137		15.273
	minimum	28		27		21		27		23			22		20		20
	median	53		48		62		64		56.5			49		56		58
	maximum	73		89		90		90		84			85		82		90
		N	%	N	%	N	%	N	%	N	%	N	%	N	%	N	%
**sex**	**m**	8	53.3%	5	55.6%	39	24.8%	20	40.8%	7	50.0%	15	60.0%	74	41.1%	168	37.4%
	**w**	7	46.7%	4	44.4%	118	75.2%	29	59.2%	7	50.0%	10	40.0%	106	58.9%	281	62.6%
**cardiovascular comorbidites**		3	21.4%	4	44.4%	74	50.3%	17	34.7%	8	61.5%	8	33.3%	62	37.6%	176	41.8%
**history of oncological illness**		2	14.3%	3	33.3%	32	21.8%	48	98.0%	4	30.8%	5	20.8%	28	17.0%	122	29.0%
**hemostaseological abnormalities**		0	0.0%	0	0.0%	8	5.4%	0	0.0%	0	0.0%	0	0.0%	5	3.0%	13	3.1%
**metabolic diseases**		3	21.4%	1	11.1%	31	21.1%	7	14.3%	4	30.8%	1	4.2%	18	10.9%	65	15.4%
**nicotine/alcohol/drug abuse**		3	21.4%	1	11.1%	19	13.1%	4	9.1%	0	0.0%	3	13.0%	18	10.9%	48	11.6%
**preoperative radiotherapay**		0	0.0%	0	0.0%	3	1.9%	5	10.2%	0	0.0%	6	24.0%	15	8.3%	29	6.5%
**preoperative chemotherapy**		0	0.0%	0	0.0%	0	0.0%	1	2.0%	0	0.0%	1	4.0%	0	0.0%	2	0.4%
**Preoperative clinical characteristics**																	
	** **	N	%	N	%	N	%	N	%	N	%	N	%	N	%	N	%
**no symptoms**		2	13.3%	0	0.0%	17	10.8%	4	8.3%	0	0.0%	1	4.0%	5	2.8%	29	6.5%
**headache**		4	26.7%	6	66.7%	33	21.0%	21	43.8%	2	14.3%	8	32.0%	22	12.2%	96	21.4%
**vertigo**		8	53.3%	4	44.4%	69	43.9%	31	64.6%	5	35.7%	11	44.0%	97	53.9%	225	50.2%
**nausea/emesis**		2	13.3%	1	11.1%	12	7.6%	18	37.5%	2	14.3%	6	24.0%	14	7.8%	55	12.3%
**cerebellar dysfunction**		0	0.0%	1	11.1%	22	14.0%	22	45.8%	2	14.3%	11	44.0%	14	7.8%	72	16.1%
**gait disturbance**		4	26.7%	1	11.1%	43	27.4%	16	33.3%	2	14.3%	5	20.0%	39	21.7%	110	24.6%
**hydrocephalus**		1	6.7%	0	0.0%	18	11.5%	9	18.4%	0	0.0%	6	24.0%	10	5.6%	44	9.8%
**visual impairment**		1	6.7%	0	0.0%	7	4.5%	1	2.1%	1	7.1%	1	4.0%	2	1.1%	13	2.9%
**hemiparesis**		0	0.0%	0	0.0%	4	2.5%	0	0.0%	0	0.0%	2	8.0%	2	1.1%	8	1.8%
**psychomotoric impairment**		0	0.0%	0	0.0%	5	3.2%	5	10.4%	1	7.1%	3	12.0%	1	0.6%	15	3.3%
**dysgeusia**		0	0.0%	0	0.0%	2	1.3%	1	2.1%	3	21.4%	1	4.0%	7	3.9%	14	3.1%
**seizures**		1	6.7%	0	0.0%	4	2.5%	2	4.2%	0	0.0%	0	0.0%	0	0.0%	7	1.6%
**trigeminal hypesthesia V1**		2	13.3%	0	0.0%	10	6.4%	0	0.0%	1	7.1%	2	8.0%	14	7.8%	29	6.5%
**trigeminal hypesthesia V2**		4	26.7%	0	0.0%	15	9.6%	1	2.1%	4	28.6%	3	12.0%	24	13.3%	51	11.4%
**trigeminal hypesthesia V3**		3	20.0%	0	0.0%	10	6.4%	0	0.0%	3	21.4%	3	12.0%	23	12.8%	42	9.4%
**facial pain**		0	0.0%	0	0.0%	15	9.6%	0	0.0%	1	7.1%	2	8.0%	2	1.1%	20	4.5%
**ocular motility disorder**		3	20.0%	0	0.0%	13	8.3%	0	0.0%	0	0.0%	5	20.0%	2	1.1%	23	5.1%
**facial nerve palsy (HB grade 2)**		0	0.0%	0	0.0%	5	3.2%	1	2.1%	1	7.1%	0	0.0%	18	10.0%	25	5.6%
**facial nerve palsy (HB grade 3)**		0	0.0%	0	0.0%	2	1.3%	2	4.2%	1	7.1%	2	8.0%	7	3.9%	14	3.1%
**facial nerve palsy (HB grade 4)**		1	6.7%	0	0.0%	1	0.6%	1	2.1%	0	0.0%	0	0.0%	4	2.2%	7	1.6%
**facial nerve palsy (HB grade 5)**		0	0.0%	0	0.0%	0	0.0%	0	0.0%	1	7.1%	0	0.0%	0	0.0%	1	0.2%
**facial nerve palsy (HB grade n.a.)**		0	0.0%	0	0.0%	1	0.6%	1	2.1%	0	0.0%	0	0.0%	0	0.0%	2	0.4%
**facial nerve palsy total**		1	1.3%	0	0.0%	9	5.7%	5	10.4%	3	21.4%	2	8.0%	29	16.1%	49	10.9%
**hypoacusia**		4	26.7%	1	11.1%	28	17.8%	4	8.3%	6	42.9%	3	12.0%	147	81.7%	193	43.1%
**tinnitus**		5	33.3%	0	0.0%	11	7.0%	2	4.2%	2	14.3%	2	8.0%	50	27.8%	72	16.1%
**cranial nerves IX/X palsy**		0	0.0%	0	0.0%	16	10.2%	1	2.1%	0	0.0%	2	8.0%	3	1.7%	22	4.9%
**cranial nerve XII palsy**	** **	0	0.0%	0	0.0%	4	2.5%	0	0.0%	2	14.3%	0	0.0%	2	1.1%	8	1.8%
**hospital stay (days)**	** **	**epidermoid cyst (N=15)**		**hemangioblastoma (N=9)**		**meningioma (N=157)**		**metastasis (N=49)**		**non-vestibular schwannomas (N=14)**		**others (N=25)**		**vestibular schwannoma (N=180)**		**total (N=449)**	
	mean	8.73		9.56		13.54		17.51		16.86			17.96		11.82		13.39
	SD	4.464		3.941		13.845		15.575		17.858			18.415		7.327		12.162
	minimum	3		5		3		5		5			5		4		3
	median	8		8		10		12		11.5			13		9		10
	maximum	16		17		133		70		76			97		47		133
**follow-up (months)**	mean	35.733		15.278		30.739		5.796		38.536			19.62		32.623		28.253
	SD	43.3119		18.1678		36.5629		14.1012		49.4942			37.1739		35.6298		35.7965
	minimum	0		0		0		0		0			0		0		0
	median	12		4.5		15		0		16			1.5		17.25		12
	maximum	131.5		45		135		87.5		139			130.5		150		150
**Preopoerative KPSS**	mean	92.67		91.11		88.13		81.04		85			79.2		88.86		87.26
	SD	9.612		9.28		13.901		11.713		10.919			22.898		12.867		13.964
	minimum	70		70		20		50		70			20		20		20
	median	100		90		90		80		85			90		90		90
	maximum	100		100		100		100		100			100		100		100

### Tumor Entities, Extent of Resection, and Surgery-Related Findings

An RSA was performed to resect 15 (3.3%) epidermoid cysts, 9 (2.0%) hemangioblastomas, 157 (35.0%) meningiomas, 49 (10.9%) metastases, 14 (3.1%) non-vestibular schwannomas, 180 (40.1%) vestibular schwannomas, and 25 (5.6%) other oncological lesions (**Table 1**).

The median approach surface was 7.8 cm^2^. A total of 292 (65.3%) osteoclastic and 155 (34.7%) osteoplastic craniotomies were performed. The median tumor volume was 5.9 cm^3^, and the gross total resection (GTR) rate was 78.8%. **Table 2** provides detailed tumor- and approach-related information and measurements as well as the extent of resection.

**Table 2:** Tumor- and approach-related characteristics and the extent of resection.

Tumor characteristics		epidermoid cyst (N=15)		hemangio-blastoma (N=9)		meningioma (N=157)		metastasis (N=49)		non-vestibular schwannomas (N=14)		others (N=25)		vestibular schwannoma (N=180)		total (N=449)	
tumor volume (cm³)	mean	20.3		16.9		16.1		16.4		12.7		8.6		6.1		11.9	
	SD	21.5		17.3		17.4		16.5		22.1		9.5		7.6		15.0	
	minimum	1.2		0.77		0.2		0.7		0.5		0.1		0.1		0.1	
	median	13.2		12.9		8.6		13		7.2		4		3		5.9	
	maximum	79.4		49.9		74.4		99.1		81.1		38.5		38.3		99.1	
hannover classification														N	%		
	T1													12	6.8%		
	T2													25	14.2%		
	T3a													27	15.3%		
	T3b													37	21.0%		
	T4a													27	15.3%		
	T4b													48	27.3%		
recurrence		N	%	N	%	N	%	N	%	N	%	N	%	N	%	N	%
		2	13.3%	1	11.1%	5	3.2%	2	4.1%	0	0.0%	3	12.0%	12	6.7%	25	5.6%
**Approach characteristics**		**epidermoid cyst (N=15)**		**hemangio-blastoma (N=9)**		**meningioma (N=157)**		**metastasis (N=49)**		**non-vestibular schwannomas (N=14)**		**others (N=25)**		**vestibular schwannoma (N=180)**		**total (N=449)**	
craniotomy surface (mm²)	mean	798.2		733.1		830.5		904.6		900.8		823.4		748.3		807.6	
	SD	174.4		292.1		273.5		325.1		210.8		339.0		247.9		274.9	
	minimum	540		342		285		416		668		387		316		285	
	median	872		656		806		823		843		735		730		776	
	maximum	1036		1153		1921		1689		1448		1522		1612		1921	
		N	%	N	%	N	%	N	%	N	%	N	%	N	%	N	%
type of craniotomy	osteoclastic	10	66.7%	2	22.2%	105	66.9%	23	46.9%	13	92.9%	14	56.0%	125	70.2%	292	65.3%
	osteoplastic	5	33.3%	7	77.8%	52	33.1%	26	53.1%	1	7.1%	11	44.0%	53	29.8%	155	34.7%
**Extent of resection**		**epidermoid cyst (N=15)**		**hemangio-blastoma (N=9)**		**meningioma (N=157)**		**metastasis (N=49)**		**non-vestibular schwannomas (N=14)**		**others (N=25)**		**vestibular schwannoma (N=180)**		**total (N=449)**	
simpson grade		N	%	N	%	N	%	N	%	N	%	N	%	N	%	N	%
	I					42	26.8%										
	II					78	49.7%										
	III					21	13.4%										
	IV					16	10.2%										
GTR (+ Simpson I/II)		11	73.3%	9	100%	120	76.4%	39	79.6%	10	71.4%	18	72.0%	141	78.3%	354	78.8%

### Postoperative Outcome, Surgical Complications, and Approach-Related Findings

The median Clavien–Dindo grade was 2, and the complication rate was 28.7% (**Table 3**). The most common complications were CSF leaks (8.6%), meningitis (5.3%), hydrocephalus (4.0%), and hemorrhage (2.7%).

**Table 3:** Postoperative complications and Clavien–Dindo Classification (CDC).

Postoperative complications		epidermoid cyst (N=15)			hemangioblastoma (N=9)			meningioma (N=157)			metastasis (N=49)			non-vestibular schwannomas (N=14)			others (N=25)			vestibular schwannoma (N=180)			total (N=449)		
		N	%	revision	N	%	revision	N	%	revision	N	%	revision	N	%	revision	N	%	revision	N	%	revision	N	%	revision
hemorrhage		0	0.0%		0	0.0%		6	4.2%	4 66,7%	0	0.0%		0	0.0%		0	0.0%		6	3.9%	5 83,3%	12	2.7%	10 76,9%
	resection cavity	0	0.0%		0	0.0%		3	50.0%		0	0.0%		0	0.0%		0	0.0%		4	66.7%		7	1.6%	
	approach	0	0.0%		0	0.0%		3	50.0%		0	0.0%		0	0.0%		0	0.0%		2	33.3%		5	1.1%	
CSF leckage/ -fistula		1	7.1%		0	0.0%		8	5.6%	6 75,0%	4	9.5%	100%	2	15.4%		1	4.8%	100%	18	11.7%	13 72,2%	34	7.6%	24 (70,6%)
wound healing disorders		1	7.1%	100%	0	0.0%		5	3.5%	3 60,0%	4	9.5%	100%	0	0.0%		1	4.8%		7	4.5%	4 57,1%	18	4.0%	12 (66,7%)
abscess		0	0.0%		0	0.0%		1	0.7%	100%	2	4.8%	100%	0	0.0%		0	0.0%		1	0.6%	100%	4	0.9%	3 (75,0%)
hydro-cephalus		0	0.0%		0	0.0%		7	4.9%		1	2.4%		2	15.4%		2	9.5%		4	2.6%		16	3.6%	
	shunt ?	0	0.0%		0	0.0%		6	85.7%		0	0.0%		2	100%		2	100%		3	75.0%		13	2.9%	
sinus vein thrombosis		1	7.1%		0	0.0%		6	4.2%		0	0.0%		0	0.0%		0	0.0%		3	1.9%		10	2.2%	
meningitis		1	7.1%		0	0.0%		10	7.0%		0	0.0%		0	0.0%		1	4.8%		9	5.8%		21	4.7%	
death		0	0.0%		0	0.0%		3	2.1%		0	0.0%		0	0.0%		0	0.0%		2	1.3%		5	1.1%	
ischemia		0	0.0%		1	11.1%		4	2.8%		2	4.8%		0	0.0%		1	4.8%		1	0.6%		9	2.0%	
**Clavien-Dindo-Classifikation**		**epidermoid cyst**			**hemangioblastoma**			**meningioma**			**metastasis**			**non-vestibular schwannomas**			**others**			**vestibular schwannoma**			**total**		
		**N (%)**			**N (%)**			**N (%)**			**N (%)**			**N (%)**			**N (%)**			**N (%)**			**N (%)**		
CDC I		0 (0,0%)			1 (100%)			6 (18,8%)			0 (0,0%)			3 (75,0%)			0 (0,0%)			4 (13,3%)			14 (18,0%)		
CDC II		3 (100%)			0 (0,0%)			12 (37,5%)			3 (50,0%)			1 (25,0%)			0 (0,0%)			10 (33,3%)			29 (37,2%)		
CDC IIIa		0 (0,0%)			0 (0,0%)			1 (3,1%)			2 (33,3%)			0 (0,0%)			0 (0,0%)			1 (3,3%)			4 (5,1%)		
CDC IIIb		0 (0,0%)			0 (0,0%)			4 (12,5%)			1 (16,70%)			0 (0,0%)			0 (0,0%)			11 (36,7%)			16 (20,5%)		
CDC IV		0 (0,0%)			0 (0,0%)			6 (18,8%)			0 (0,0%)			0 (0,0%)			2 (100%)			2 (6,7%)			10 (12,8%)		
CDC V		0 (0,0%)			0 (0,0%)			3 (9,4%)			0 (0,0%)			0 (0,0%)			0 (0,0%)			2 (6,7%)			5 (6,4%)		

Regarding the patient population, statistical analysis showed no significant correlation between metabolic diseases and postoperative wound healing disorders (p = 0.165) or abscesses (p = 0.479). In addition, we found no significant correlation in patients with preoperative nicotine/alcohol/drug abuse (p = 0.26 and p = 0.413, respectively). No significant correlation between hemostaseological disorders and postoperative hemorrhage could be identified (p = 1.0). Furthermore, no significant correlation between the previous radiotherapy and wound healing disorders (p = 1.00), a surgical revision (p = 0.556), or abscesses (p = 1.00) could be detected.

Findings revealed that tumor volume had no significant impact on postoperative complications in general (p = 0.086) but had a significant impact on postoperative hemorrhage (p = 0.037) and hydrocephalus (p = 0.019). Tumor volume was significant for several preoperative symptoms (p < 0.001). Also, in the case of meningiomas and vestibular schwannomas, tumor volume had a significant influence on hydrocephalus (p = 0.01) and meningitis (p = 0.025). Interestingly, there was a significant correlation between the Simpson grade and tumor volume (p = 0.001); i.e., tumor volume affected the extent of resection (ρ < 0.5) in the case of meningiomas. Increasing tumor volume led to more extended craniotomies (p = 0.003) and vice versa (ρ < 0.5).

The extent of the approach had no significant impact on complications in general (p = 0.120) but was significant regarding postoperative CSF leaks (p = 0.008). No significant correlation between craniotomy technique (osteoplastic/osteoclastic) and postoperative complications was found (p = 0.209). Craniotomy size was not significant regarding GTR (p = 0.178), and GTR rate had a borderline significant correlation with tumor volume (p = 0.056); in the case of vestibular schwannomas, size of craniotomy was important for GTR (p = 0.041).

## Discussion

Findings revealed that tumor volume had no significant impact on postoperative complications in general (p = 0.086) but had a significant impact on postoperative hemorrhage (p = 0.037) and hydrocephalus (p = 0.019). Tumor volume was significant for several preoperative symptoms (p < 0.001). The extent of the approach had no significant impact on complications in general (p = 0.120) but was significant regarding postoperative CSF leaks (p = 0.008). Craniotomy size was not significant regarding GTR (p = 0.178). In the case of vestibular schwannomas, size of craniotomy was important for GTR (p = 0.041).

The complication rate was 28.7%, which is a satisfactory result as compared to previous experiences (1–4, 7–10, 15–19).

However, the approach-related obstacles and complications must be taken into consideration when choosing the RSA; anatomic approach-related knowledge is needed to avoid unnecessary increasing complication rates. Direct comparison to the findings will remain difficult considering that others focused on either specific entities or vascular lesions and less on the approach.

### The Right Approach—A Needle in the Haystack?

For decades, several authors have described alternative more or less complicated and demanding approaches to reach the petroclival region or the CPA as an alternative to the prominent RSA. The RSA has undergone several modifications and extensions.

Regarding the petroclival region, numerous reports detailed the variety of cranial base approaches that can be used (2, 7, 20–22). In 1973, Morrison et al. described a combined transtentorial approach through performing a translabyrinthine petrosectomy (23). Later reports contained descriptions of modern combined transpetrosal approaches to preserving the sigmoid sinus (24, 25). The advantages of the combined approaches include a wide surgical field, multiple axes of dissection, minimal brain retraction, and early access to feeding vessels. However, major disadvantages are increased risk of damage to the facial nerve, temporal lobe retraction, increased risk of injury to the vein of Labbé, and increased operative time.

For example, in a series by Erkmen et al. with 97 patients, their choice of approach depended on the tumor’s location along the clivus and its relation to the internal auditory meatus (IAM) (26). They used an orbitozygomatic approach to resect tumors medial to the IAM, whereby a posterior transtentorial petrosal approach was performed for tumors located laterally to the IAM. An anterior petrosectomy was additionally performed to treat extensive tumors growing into the middle cranial fossa and cavernous sinus. These exemplary reports display the complexity and the desire for a successful surgical outcome. Many studies emphasize the desire and the goal to preserve patients’ hearing and facial nerve function; Sekhar et al. added a partial labyrinthectomy to a standard presigmoid petrosal approach to treat 25 patients with petroclival meningiomas with a hearing preservation rate of 81%. Regardless, 47% suffered from new cranial nerve deficits postoperatively (27). The translabyrinthine and transcochlear approaches were used in 47% of patients. However, the proportion of patients in which hearing preservation was a primary concern increased substantially over time; the authors concluded that patients should undergo surgery to preserve hearing.

Others have used alternative approaches, particularly the RSA, with success (8–10, 28–30). Bricolo et al. emphasized that the consistency and degree of neurovascular encasement of the tumor are major determinants of the degree of safe resection that can be achieved (31).

Cadaveric studies demonstrate a similarity between the working area the RSA provides and combined petrosal approaches to the petroclival surface without including a complete transcochlear exposure; there is no significant difference between these approaches in either the working area or the angle of attack to the petroclival surface.

The RSA provides an equivalent working area and angles of attack for petroclival lesions compared to a combined transpetrosal approach (32). In addition, the RSA provides a significantly larger clival and brainstem working area compared to Kawase’s approach (19). Although using cerebellar retraction is a potential risk factor for intraoperative edema and cerebellar infarction, we did not experience severe postoperative consequences.

Our findings revealed that craniotomy size was not significant regarding GTR (p = 0.178), and GTR rate barely missed a significant correlation with tumor volume (p = 0.056). On the other side, measurements revealed that increasing tumor volume led to more extended craniotomies (p = 0.003).

Of course, the petrous apex can be approached using Kawase’s approach, but the labyrinth limits it laterally, and the petrous internal carotid artery limits it anterolaterally. The approach cannot provide access below the level of the IAM. As an extension of Kawase’s approach and a modification of the Goel technique, Morisako et al. described a middle skull base approach with posterolateral mobilization of the geniculate ganglion to access the clival region (1). Morisako’s and Goel’s techniques are middle fossa transcochlear approaches with the drilling of the cochlea and mobilization of the facial nerve. Regardless, as mentioned above, they are hearing-sacrificing ones.

Due to these problems, the classic RSA was modified to access the compartment of the middle fossa *via* the infratentorial space by drilling the portion of the petrous bone that is located anterior to and above the IAM (1).

Other authors reported similar techniques (7). They described the surgical technique of a retrosigmoid craniotomy followed by drilling between the 7/8th cranial nerve complex and the 5th cranial nerve. Most of the reports advocate osteoclastic techniques, whereby no significant correlation between craniotomy technique (osteoplastic/osteoclastic) and postoperative complications was found (p = 0.209) in our series. Thus, the technical decision may depend on institutional experience and standards. The resected bone area, in a particular sense, is similar to the area exposed extradurally through Kawase’s approach (11). By using a modified RSA, the direction from which the bone area is drilled is posterior and below, whereas using Kawase’s approach, it is anterior and above. Dumbbell-shaped trigeminal schwannomas, for example, usually expand the bone surrounding Meckel’s cave, creating an enlarged space between the posterior and middle fossae. This space is accessed using an RSA by drilling away the suprameatal bone. The retrosigmoid route provides early visualization of cranial nerves. The presence of large tumors increases the working space within the CPA. As the majority of tumors displace the 7th–8th cranial nerve complex downward, and in cases of meningiomas with a matrix at the petrous apex, the 5th nerve upward, the usually narrow anatomical space is enlarged (9, 23, 33). In the majority of cases, the tumor displaces and/or compresses the Dandy vein; collateral veins have developed so that the petrosal vein usually no longer represents an obstacle. A further advantage of the RSA is the possibility of mobilizing the 5th nerve after opening Meckel’s cave, thus improving the chances of preserving it. In addition, earlier identification of the 6th nerve at the brainstem during tumor dissection is enabled, unlike with the lateral transpetrosal approaches.

Currently, the RSA remains the gold standard for pathologies in the CPA; the conventional technical notes have been described previously in detail (1–4, 8–10, 14, 17, 34, 35). The RSA is the most commonly used approach in removing vestibular schwannomas or CPA meningiomas. Some still advocate the translabyrinthine approach with its good visualization with minimal cerebellar retraction (18, 36, 37). However, in addition to being a hearing-destructive technique, it is associated with a high incidence of CSF leaks. The retrosigmoid technique, although requiring cerebellar retraction and having limitations in gaining lateral exposure of the IAC, provides sufficient exposure to the CPA.

Some series report that tumor size significantly correlates with the possibilities for total removal, anatomical and functional facial nerve preservation, and the rate of complications (18, 33, 38). However, we could not find a significant correlation between size and GTR, keeping our heterogeneity in mind. In our series, increasing tumor volume led to more extended craniotomies (p = 0.003).

We could demonstrate that conventional approach techniques and bigger craniotomies should not always be criticized, as decent outcomes are feasible.

It is not negligible that several factors regarding GTR are independent of the particular surgical approach chosen or even of the surgical team’s skill or experience. These factors have been well described and include cavernous sinus invasion, brainstem pial invasion, neurovascular structure involvement, and tumor consistency. Factors such as vicinity to the IAC, involvement of one or both cranial fossae, and preoperative hearing functional status are critical considerations in determining the optimal strategy for treating all the pathologies in the posterior fossa.

### Study Limitations

As this is a retrospective case series, it is not possible to draw causalities to clinical outcomes. Nevertheless, we implemented detailed clinical examination, including scores on functional performance, and a standardized follow-up protocol based on a certified neurooncological board into our clinical workflow. That said, the current study has some noteworthy limitations. In addition to its retrospective nature, the analyzed patients collectively suffer from certain aspects of heterogeneity regarding pathology and tumor entity. We decided to include all treated pathologies by focusing more on approach-related findings and less on the oncological outcomes. Aiming at the approach-related complications, we decided to focus not only on lesions but also on neurovascular compression syndromes because approach techniques were similar; thus, it is possible that complications could be reduced on the approaches as well. Despite the heterogeneity of the oncological lesions, another limitation is the number of individual surgeons involved in this study. However, regarding the size of the approach, it was interesting to have several surgeons involved in this analysis; like personal history, experience and technique may favor a bigger or smaller craniotomy, as there has been no real gold standard yet. Thus, we decided not to filter the number of surgeons. Of course, only experienced surgeons have performed the surgical procedures, but by analyzing as many approaches as possible, we have been able to have access to the different craniotomy sizes. However, the significant results have to be treated with caution.

## Conclusion

Tumor volume has an important impact on the preoperative symptoms as well as on postoperative complications. Although the extent of the craniotomy barely missed significance regarding GTR, a correlation can be assumed. Thus, the extent of craniotomy should be taken into presurgical consideration, especially in the case of postoperative CSF leaks. Regarding vestibular schwannomas, craniotomy size plays an important role to achieve satisfactory oncological outcomes. Different approaches should be selected where necessary with superior resection rates.

## Data Availability Statement

The raw data supporting the conclusions of this article will be made available by the authors, without undue reservation.

## Author Contributions

Conceptualization: AA and AJ. Methodology: AA, AJ, FH, and SH. Formal analysis and investigation: AA, AJ, and SH. Writing—original draft preparation: AA. Writing—review and editing: AA, AJ, SH, FH, BW, DB, SC, BM, CN, and JG. Supervision: BW, DB, SC, CN, BM, and JG. All authors listed have made a substantial, direct, and intellectual contribution to the work and approved it for publication.

## Conflict of Interest

JG and BM work as consultants for Brainlab (Brainlab AG, Feldkirchen). In addition, BM works as a consultant for Medtronic, Spineart, Icotec, Relievant, and DePuy/Synthes. In these firms, BM acts as a member of the advisory board. Furthermore, BM reports a financial relationship with Medtronic, Ulrich Medical, Brainlab, Spineart, Icotec, Relievant, and DePuy/Synthes. He received personal fees and research grants for clinical studies from Medtronic, Ulrich Medical, Brainlab, Icotec, and Relievant. All this happened independently of the submitted work. BM holds royalties/patents for Spineart. All named potential conflicts of interest are unrelated to this study.

The remaining authors declare that the research was conducted in the absence of any commercial or financial relationships that could be construed as a potential conflict of interest.

## Publisher’s Note

All claims expressed in this article are solely those of the authors and do not necessarily represent those of their affiliated organizations, or those of the publisher, the editors and the reviewers. Any product that may be evaluated in this article, or claim that may be made by its manufacturer, is not guaranteed or endorsed by the publisher.
